# Amino Acid Utilization During Twelve Consecutive Industrial Repitching Cycles of *Saccharomyces pastorianus* in Lager Beer Fermentation

**DOI:** 10.3390/foods15193373

**Published:** 2026-09-22

**Authors:** Ksenija Obrovnik, Miha Ocvirk, Nataša Kočar Mlinarič, Iztok Jože Košir

**Affiliations:** 1 Department for Agrochemistry and Brewing, Slovenian Institute of Hop Research and Brewing, Cesta Žalskega Tabora 2, 3310 Žalec, Slovenia; ksenija.obrovnik@ihps.si (K.O.); miha.ocvirk@ihps.si (M.O.); 2 Biotechnical Faculty, University of Ljubljana, Jamnikarjeva 101, 1000 Ljubljana, Slovenia; kocar.natasa@gmail.com

**Keywords:** lager beer, brewing yeast, serial repitching, *Saccharomyces pastorianus*, amino acids, yeast reuse, industrial fermentation

## Abstract

Amino acids are key assimilable nitrogen sources for brewing yeast and important precursors of beer flavor compounds. This study investigated amino acid utilization during 12 consecutive industrial lager fermentations with *Saccharomyces pastorianus* under serial repitching conditions. Samples were collected throughout fermentation and analyzed by HPLC. Fermentation performance remained comparatively stable across all repitching cycles, with little variation in final alcohol content, real degree of fermentation, apparent extract, and pH. Later cycles reached comparable terminal states approximately one day earlier. Exploratory principal component analysis showed that PC1 accounted for 75.29% of total amino-acid-profile variance and primarily reflected the progression of samples from early to later fermentation stages; principal components were not assigned directly to experimental factors. Terminal-value regressions identified the strongest cycle-associated trend for aspartate (nominal *p* < 0.001; adjusted R^2^ = 0.71), which remained statistically supported after false-discovery-rate correction. Nominal trends observed for asparagine and serine did not remain significant after correction for multiple testing. Overall, conventional fermentation performance and the global structure of amino acid utilization remained comparatively stable across the twelve-cycle industrial sequence. Because the experiment comprised a single consecutive repitching sequence rather than independently replicated generations, cycle-associated changes should be regarded as exploratory process-level observations.

## 1. Introduction

Amino acids represent the principal assimilable nitrogen source for brewing yeast and are fundamental determinants of fermentation performance, yeast growth, and beer flavor development [1,2]. Together with ammonium and small peptides, they constitute free amino nitrogen (FAN), which supports biomass formation and contributes directly to the biosynthesis of higher alcohols, esters, carbonyl compounds, and sulfur-containing metabolites. Because these metabolites strongly influence sensory quality, the composition and utilization of wort amino acids are central to both process efficiency and flavor outcomes in brewing [3,4,5].

Yeast does not consume wort amino acids randomly; rather, uptake follows a structured hierarchy. Amino acids such as asparagine, aspartate, serine, threonine, glutamate, lysine, arginine, and often methionine are typically assimilated rapidly and early during fermentation, indicating their preferential use in nitrogen metabolism. In contrast, many other amino acids, including the branched-chain amino acids, are taken up more gradually, while glycine and alanine are usually consumed later, after the preferred pool has been depleted. Proline is characteristically poorly utilized under standard brewing conditions and often remains largely unconsumed in finished beer [6]. However, the exact ranking and grouping of amino acids according to their assimilation order can vary among different authors, reflecting differences in experimental conditions, yeast strains, and classification approaches [3,4,6,7,8,9].

Beyond their role as nitrogen sources, specific amino acids act as important flavor precursors and regulators of yeast metabolism. Branched-chain and aromatic amino acids, together with glutamate, serine, lysine, and histidine, have particularly strong effects on the formation of higher alcohols and esters [3,5,10]. Even relatively small changes in amino acid availability can alter fermentation degree, rebalance nitrogen regulatory pathways such as nitrogen catabolite repression (NCR), amino acid sensing pathway (SPS), and target of rapamycin pathway (TOR), and modify the production of aroma-active metabolites [5,10,11]. Moreover, brewing yeast co-utilizes ammonia, free amino acids, and peptides, with peptides contributing substantially to the total protein nitrogen pool. Consequently, shifts in the balance between peptide uptake and free amino acid assimilation may influence nitrogen efficiency and flavor precursor availability even when total FAN levels appear similar [12,13].

The repeated reuse of yeast in brewery practice introduces an additional layer of complexity. Serial repitching is widely employed for economic and technological reasons, but repeated cropping and re-inoculation can progressively reshape yeast physiology and nitrogen metabolism. Industrial-scale studies have demonstrated that serial repitching can modify substrate utilization dynamics without compromising overall fermentation performance. For example, successive yeast reuse was shown to enhance the efficiency of wort saccharide assimilation while maintaining high yeast viability and fermentation quality, suggesting that adaptive physiological changes occur during repeated industrial fermentations [14]. Since carbon and nitrogen metabolism are tightly interconnected during yeast growth, similar adaptive responses may also influence amino acid assimilation. Previous studies have shown that the effects of serial repitching are not necessarily uniform across all amino acids. In some worts, only a subset of amino acids displays clear cycle associated responses, whereas overall assimilation remains robust, indicating that serial reuse does not inevitably lead to a general collapse of nitrogen uptake. In other substrates, however, amino acid assimilation may become increasingly disturbed after several fermentation cycles, suggesting the emergence of systemic metabolic stress and practical limits to yeast reuse. These findings indicate that the consequences of repitching are strongly substrate-dependent and may selectively affect the timing, extent, and sensitivity of individual amino acid uptake patterns [1,8].

From a brewing perspective, such changes are highly relevant because altered amino acid utilization has the potential to influence both yeast vitality and the precursor pool for higher alcohol and ester formation. However, the extent to which such biochemical changes translate into measurable sensory differences remains unclear, particularly under controlled industrial conditions. The present study therefore investigated amino acid utilization during 12 successive repitching cycles of *Saccharomyces pastorianus* under industrial-scale lager brewing conditions. The objective was to determine whether serial repitching alters stage-dependent amino acid depletion patterns, whether these effects are amino-acid-specific rather than global, and to assess whether such biochemical changes are accompanied by observable deviations in overall sensory quality under industrial conditions.

## 2. Materials and Methods

### 2.1. Brewing Process and Experimental Design

Industrial-scale lager beer production was conducted in cylindroconical fermentation vessels (total volume 4400 hL, working volume 3250 hL) (Ziemann, Ludwigsburg, Germany) using a conventional brewing protocol. Raw materials (malt, corn, and hops) were sourced under controlled conditions to ensure batch-to-batch consistency. The brewing protocol, fermentation conditions, and repitching strategy were identical to those described previously for the investigation of carbohydrate utilization during successive industrial fermentations [14].

The same yeast culture—bottom-fermenting lager yeast strain, *Saccharomyces pastorianus* TUM 34/70 (Weihenstephan Yeast Centre, Freising, Germany)—was reused across 12 consecutive fermentation cycles (A–L). The twelve fermentations constituted one consecutive industrial repitching sequence rather than independently replicated fermentations at each repitching generation. Accordingly, repitching cycle was inherently confounded with chronological fermentation order. Observed changes across cycles therefore represent within-process, cycle-associated trends and cannot by themselves distinguish the biological effects of repeated yeast reuse from temporal or process-related drift. After each primary fermentation, yeast biomass was harvested, stored at 4 °C for 48 h without agitation, and subsequently repitched into fresh wort under comparable process conditions.

### 2.2. Sampling Procedure

Wort samples were collected immediately after wort production, stored at 0 °C, and frozen within 24 h. During fermentation, samples were taken at approximately 24 h intervals (±30 min). Immediately after sampling, sodium azide (final concentration 0.02 g/L) was added to inhibit further metabolic activity and prevent post-sampling changes in amino acid concentrations. Samples were centrifuged (4000× *g*, 10 min) to remove yeast biomass, and supernatants were stored frozen until analysis. Final beer samples were collected after maturation, representing the finished product, and were analyzed under the same analytical conditions. A total of 6–8 sampling points per fermentation were obtained, depending on fermentation duration. Prior to analysis, samples were thawed at room temperature, degassed using an ultrasonic bath, and filtered through cellulose acetate membrane filters (0.45 µm). Due to differences in fermentation kinetics, earlier repitching cycles (A–E) contained one additional intermediate sampling point compared to later cycles (F–L).

### 2.3. Methods

The samples were analyzed employing the methods of the European Brewing Convention (EBC) (issued in Analytica–EBC, Analytica–Microbiologica–EBC and MEBAK). The samples were degassed by shaking for 10 min at 150 rpm on a shaker (HS 50; IKA-Werke, Staufen, Germany). The samples were then filtered through a Whatman grade 597½ filter, diameter 240 mm, pore size 4–7 μm (particle retention) (Whatman, Buckinghamshire, UK).

Determination of the real, original and apparent extract and original gravity were made on an SP-1m Beer Analyzer (Anton Paar, Graz, Austria) instrument according to the MEBAK method [15]. Alcohol content was determined according to Analytica–EBC method 9.2.6 [16] using an SP-1m Beer Analyzer (Anton Paar, Graz, Austria) instrument. The real degree of fermentation (RDF) was determined according to Analytica–EBC method 9.5 [17] and calculated from the measurements of the alcohol content and real extract using the following equation:

(1) $$RDF=(\frac{2.0665\cdot w\left(A\right)}{2.0665\cdot w(A)+w{(E}_{R})})\cdot 100$$

where *w*(A) is the alcohol in %, *w*(E_R_) is the real extract in %, and 2.0665 is a numerical factor.

The wort and beer pH values were determined at 20 °C according to Analytica–EBC method 9.35 [18] using a pH meter (MPC227; Mettler Toledo, Urdorf, Switzerland) with an InLab Export Pro electrode (Mettler Toledo, Switzerland).

The viability of the yeast cells was determined by methylene blue staining according to Analytica–Microbiologica–EBC method 3.2.1.1 [19], using a 40× microscope magnification (BH2; Olympus, Tokyo, Japan).

Amino acids were analyzed using HPLC apparatus (Agilent 1200 Series, Agilent Technologies, Santa Clara, CA, USA) equipped with a thermostated autosampler, a reverse phase column (Agilent Eclipse PAH, 4.6 × 100 mm, 1.8 µm, Agilent Technologies, Santa Clara, CA, USA), and a fluorescence detector (FLD). The analytical procedure was based on the method optimized by the HPLC instrument manufacturer [20]. The excitation and emission wavelengths for OPA derivatives were set to 340 and 450 nm, respectively, and for Fmoc derivatives to 266 and 305 nm, respectively. The HPLC instrument control and analysis of results were conducted with ChemStation 32 computer software (Agilent Technologies, Santa Clara, CA, USA). Amino acid quantification was performed using external calibration with commercially available standards. Calibration curves were constructed for each amino acid over an appropriate concentration range (R^2^ > 0.995). Instrumental repeatability was verified by replicate injections (n ≥ 3), and relative standard deviations were below 10% for major amino acids. Sample preparation and analysis were performed under identical conditions to ensure comparability across all repitching cycles.

Sensory evaluation of all beer samples included taste, odor and bitterness rating by seven experts, who gave seven independent marks. Expert panel consisted of 3 females and 4 males, aged between 32 and 56. The evaluation took place in a separate tasting room, with room temperature from 18 to 25 °C and humidity between 50 and 70%. All beer samples were tasted fresh one week after the bottling. At the time of tasting, the temperature of the samples was between 10 and 11 °C. Evaluation marks ranged from 1 to 5 for each parameter.

### 2.4. Statistical Analysis

Statistical analyses were performed using OriginPro^®^ 2020b (OriginLab Corporation, Northampton, MA, USA). Linear mixed-effects models were fitted in Python using the statsmodels package (version 0.14.6). Amino acid concentrations were expressed relative to the corresponding wort sample of each repitching cycle, with wort set to 100%. Terminal fermentation parameters were summarized as the mean ± standard deviation across the 12 repitching cycles. Principal component analysis (PCA) was performed on normalized amino acid concentrations to evaluate the dominant sources of variation in the dataset. Linear mixed-effects models were fitted separately for each amino acid to evaluate the association between sampling day and relative amino acid concentration. Sampling day was treated as a categorical fixed effect, whereas repitching cycle was included as a random intercept to account for repeated observations within each industrial fermentation. Day 1 was used as the reference category. Overall sampling-day effects, Wald chi-square statistics, degrees of freedom, *p*-values, FDR-adjusted q-values, variance components, and intraclass correlation coefficients are reported in Supplementary Table S3. Because the study comprised a single consecutive industrial repitching sequence, the mixed-model results were interpreted as exploratory within-process associations rather than independently replicated repitching effects. Cycle-associated trends in terminal amino acid concentrations were evaluated by linear regression, with repitching cycle treated as a continuous variable from 1 to 12. Model fit for the terminal-value regressions is reported as adjusted R^2^. Adjusted R^2^ can take negative values when the fitted model explains less variation than expected relative to an intercept-only model. Because separate terminal-value regressions were performed for 20 amino acids, *p*-values were additionally adjusted for multiple testing using the Benjamini–Hochberg false discovery rate (FDR) procedure. Statistical interpretation was based primarily on the FDR-adjusted values, while nominal *p*-values are also reported for transparency. Because the experiment comprised a single serial repitching sequence, inferential statistics were used to characterize associations within this industrial process rather than to estimate independently replicated cycle associated effects. *p*-values and model coefficients should therefore be interpreted as exploratory descriptors of within-sequence trends. After sensory evaluation, OriginPro^®^ statistical software was used for statistical analysis to determine average marks and their standard deviations. A sensory mark of 4.5 to 5.0 means a very good, pleasant, well balanced beer, a perfect example of this kind of beer; 3.5 to 4.5 means a good/clean fresh beer that may have a characteristic flavor attribute at a slightly elevated or reduced level; 2.5 to 3.5 means neither a good nor poor beer with off flavors at a low level and/or an imbalance of brand flavor characteristics; 1.5 to 2.5 means a poor beer with one or more intense off flavors; and 1.0 to 1.5 means a very poor beer, undrinkable/wrong product. The main purpose of sensorial evaluation was to detect possible sensory mistakes in the produced beer to be sure that all samples were adequate.

## 3. Results

### 3.1. Fermentation Performance Across Repitching Cycles

Across the 12 consecutive repitching cycles (A–L), fermentation proceeded consistently to comparable terminal states, which is presented in Table 1. Final alcohol concentration (5.07 ± 0.05 vol%), real degree of fermentation (RDF) (83.65 ± 0.47%), apparent extract (1.87 ± 0.07%), residual sugars (0.39 ± 0.08 g/100 mL), and pH (4.31 ± 0.06) showed minimal variability and no significant cycle associated trends. Extract yield as a separate parameter was not calculated in this study. These results indicate that serial repitching did not impair the overall fermentative capacity of the yeast under the applied industrial conditions.

Fermentations in later repitching cycles (F–L) reached comparable terminal states approximately one day earlier than earlier cycles (A–E), indicating a modest acceleration of fermentation without affecting the final product composition.

Terminal yeast cell concentrations declined progressively across the twelve-cycle sequence, from 13.4 × 10^6^ cells/mL in cycle A to 4.4 × 10^6^ cells/mL in cycle L. Exploratory linear regression against cycle number yielded a slope of approximately −0.81 × 10^6^ cells mL^−1^ cycle^−1^ (R^2^ ≈ 0.82, nominal *p* < 0.001). Because cycle number was inseparable from chronological fermentation order, this relationship is interpreted as a within-sequence trend rather than as an independently replicated effect of repitching. Despite the lower terminal cell concentrations in later cycles, final alcohol content, RDF, apparent extract, and pH remained comparatively stable. Yeast viability remained above 95% across all twelve repitching cycles (Supplementary Table S2).

### 3.2. Global Structure of Amino Acid Utilization

Absolute initial amino acid concentrations for each of the twelve starting worts are provided in Supplementary Table S1. These data were retained to allow for an evaluation of between-cycle variation in initial nitrogen availability, whereas normalized concentrations were used to compare proportional depletion during fermentation. Starting-wort concentrations showed appreciable variation for several amino acids, reinforcing the use of within-wort normalization when comparing depletion patterns. Relative amino acid concentrations, normalized to the corresponding wort composition (set to 100%), exhibited a consistent depletion-driven pattern across all fermentations. PCA of the normalized amino acid profiles showed that PC1 and PC2 accounted for 75.29% and 6.99% of the total variance, respectively. Samples collected at different stages of fermentation tended to occupy different regions of the score plot, with progression from early to later sampling points occurring predominantly along PC1 (Figure 1). This pattern is consistent with progressive amino acid depletion as a major source of multivariate variation. Because PCA is unsupervised, however, the principal components were not interpreted as direct measures of fermentation stage or repitching cycle, and PC2 was not assigned to a specific experimental factor.

Linear mixed-effects modeling confirmed a strong association between sampling day and relative amino acid concentration (Supplementary Table S3). When models were fitted separately for each amino acid, with sampling day as a categorical fixed effect and repitching cycle as a random intercept, the overall effect of sampling day was statistically supported for all 20 amino acids. The strongest sampling-day associations were observed for asparagine [χ^2^(7) = 2022.28, *p* < 0.001], isoleucine [χ^2^(7) = 2015.98, *p* < 0.001], and serine [χ^2^(7) = 1477.97, *p* < 0.001]. Proline showed the weakest, although still statistically supported, association [χ^2^(7) = 22.00, *p* = 0.003]. All overall sampling-day effects remained statistically supported after Benjamini–Hochberg FDR correction (q ≤ 0.003). These results quantitatively support the temporal structure observed in the PCA and amino acid depletion profiles. Relative concentrations decreased markedly during fermentation, with the most pronounced changes occurring during early and mid-fermentation, followed by stabilization toward the terminal phase. These results are consistent with the established hierarchical uptake of nitrogen sources by brewing yeast [21].

In contrast, PCA did not reveal an obvious global separation attributable to consecutive repitching cycles, whereas terminal-value regressions identified selected cycle-associated trends at the individual-amino-acid level. Together, these findings indicate that the global structure of amino acid depletion was preserved during serial yeast reuse.

### 3.3. Amino Acid-Specific Depletion Patterns

Individual amino acids exhibited distinct and reproducible depletion behaviors consistent with known nitrogen utilization hierarchies. Figure 2 shows the relative concentrations of amino acids during sampling, at different generations. Rapid and extensive depletion relative to the corresponding initial wort concentration was observed for asparagine, serine, threonine, leucine, lysine, isoleucine, methionine, aspartate, and glutamate. Because each wort concentration was defined as 100%, the low terminal percentages of asparagine and serine indicate extensive utilization during fermentation rather than low absolute abundance. Their rapid depletion is consistent with their preferential assimilation as nitrogen sources by brewing yeast, although the present study did not investigate the specific transport or regulatory mechanisms responsible for these patterns.

These patterns were highly conserved across all repitching cycles, further indicating that the fundamental structure of amino acid utilization was maintained during serial yeast reuse. For several observations, most notably proline, normalized concentrations exceeded 100%. Because values were calculated relative to the corresponding initial wort concentration, percentages above 100% indicate that the measured free amino acid concentration at the later sampling point exceeded that measured in the starting wort. This may reflect net release or redistribution of free amino acids during fermentation, including contributions from cellular turnover or peptide/protein hydrolysis, as well as analytical and normalization variability. The present experimental design does not allow these possible sources to be distinguished.

### 3.4. Cycle-Associated Trends in Terminal Amino Acid Concentrations

To assess the potential cumulative effects of serial repitching, cycle associated trends in terminal relative amino acid concentrations were evaluated using linear regression, with repitching cycle treated as a continuous variable (1–12). Terminal amino acid concentrations [relative %] are listed in Table 2.

In Table 3, the results of linear regression are shown. Among the analyzed amino acids, aspartate showed the strongest positive cycle-associated trend in terminal relative concentration (nominal *p* < 0.001; adjusted R^2^ = 0.71). This association remained statistically supported after Benjamini–Hochberg FDR correction (q < 0.05). Asparagine (nominal *p* = 0.013) and serine (nominal *p* = 0.037) showed negative slopes at the nominal level; however, neither association remained significant after correction for multiple testing. These observations are therefore treated as exploratory tendencies rather than statistically supported cycle-associated effects. No clear cycle-associated trends were identified for the remaining amino acids.

For the majority of amino acids, including alanine, arginine, GABA, glutamate, glycine, and branched-chain amino acids, no significant generation-dependent trends were observed (*p* > 0.05), indicating that their utilization remained stable throughout serial repitching.

### 3.5. Integration of Global and Compound-Specific Effects

Taken together, these results show pronounced sampling-day-associated changes in amino acid concentrations, while only limited compound-specific cycle-associated variation was detected. The strongest cycle-associated trend was observed for aspartate, whereas most amino acids followed stable depletion trajectories across all repitching cycles.

Comparison between early (A–E) and late (F–L) repitching cycles supported this interpretation. Later cycles showed slightly higher terminal residues for selected amino acids, most notably aspartate, while others, such as asparagine and serine, tended to be more completely depleted. However, the overall magnitude of variation remained limited, and no systematic shift in global amino acid utilization was observed.

Due to the lack of independent biological replication, these findings should be interpreted as exploratory process-level trends rather than confirmatory statistical evidence.

### 3.6. Sensory Evaluation

Sensory evaluation was conducted as a qualitative quality-control assessment. No major sensory defects were detected across the repitching cycles, and all samples were considered to be within the acceptable quality range. No major sensory defects were detected in this qualitative quality-control assessment, and all samples were considered within the acceptable quality range. However, the assessment was not designed to quantify subtle sensory differences among repitching cycles.

## 4. Discussion

The present study demonstrates that the serial repitching of *Saccharomyces pastorianus* over 12 consecutive industrial scale lager fermentations does not compromise overall fermentation performance, while inducing only subtle and compound-specific changes in amino acid utilization. Core technological parameters, including alcohol content, degree of fermentation, residual extract, and pH, remained highly stable across all repitching cycles, confirming that yeast functionality was preserved within the investigated reuse window. The decline in terminal suspended yeast cell concentration is noteworthy because it occurred despite preserved final fermentation performance and slightly shorter fermentation times in later cycles. Terminal cell concentration is influenced by several processes, including growth, sedimentation/flocculation, harvesting behavior, and physiological state, and therefore cannot be interpreted directly as loss of yeast viability. The stability of alcohol production and attenuation indicates that the lower terminal suspended-cell counts did not correspond to an evident decline in overall fermentation capacity within this sequence. The observation that later repitching cycles reached terminal conditions more rapidly suggests a modest acceleration of fermentation, which may have been associated with differences in the physiological state of the yeast population. However, the present experimental design does not allow the underlying cause to be established.

A central finding of this study is that amino acid profiles changed markedly during fermentation progression, whereas no obvious global separation according to repitching cycle was observed in the PCA. Linear mixed-effects models further demonstrated statistically supported sampling-day effects for all individual amino acids. The observed depletion patterns closely followed established nitrogen utilization hierarchies, with a rapid uptake of preferred amino acids during the early fermentation and stabilization of residual concentrations at later stages. The high reproducibility of these patterns across all repitching cycles indicates that the overall organization of nitrogen assimilation was largely preserved during serial yeast reuse.

Hop-derived bitter compounds constitute an additional component of the fermentation environment that may influence microbial physiology. Iso-α-acids are well-recognized for their antimicrobial activity, particularly against Gram-positive bacteria, whereas Saccharomyces yeasts exhibit substantially greater tolerance through mechanisms including vacuolar sequestration, cell-wall adaptation, and active transport [22]. Nevertheless, hop addition was not manipulated in the present experiment, and the brewing protocol was maintained consistently throughout the twelve-cycle sequence. Therefore, the present data cannot distinguish any specific contribution of hop-derived compounds to amino acid utilization, and the observed amino acid patterns should be interpreted as responses to the complete industrial wort and fermentation environment rather than to hops specifically.

Within this comparatively stable global pattern, aspartate showed the clearest cycle-associated change, with increasing terminal relative concentrations across the A–L sequence. This pattern is consistent with progressively lower apparent depletion of aspartate in the later cycles. Several biological explanations could be hypothesized, including changes in amino-acid transport capacity, intracellular nitrogen demand, competition among nitrogen sources, or the physiological state of the repitched yeast population. However, none of these mechanisms was measured directly in the present study. The aspartate trend should therefore be regarded as an empirical process-level observation requiring mechanistic investigation rather than as evidence for a specific alteration in transporter or regulatory function. Asparagine and serine showed negative cycle-associated trends at the nominal *p* < 0.05 level; however, these associations did not remain statistically significant after false-discovery-rate correction. They should therefore be regarded as exploratory tendencies rather than evidence of reproducible changes during repitching. No additional amino acids showed statistically supported cycle-associated terminal trends after FDR correction.

The overall preservation of amino acid depletion patterns is consistent with previous observations that repeated use of lager yeast does not necessarily produce a generalized deterioration of nitrogen assimilation. Deželak et al. [8] demonstrated that the response to serial repitching was amino-acid- and substrate-dependent, with substantial differences among barley, buckwheat, and quinoa worts. The present industrial barley-based fermentation sequence similarly indicates that individual amino acids may respond differently to repeated yeast reuse while the global pattern of nitrogen utilization remains comparatively robust. This contrasts with a simple model in which progressive repitching uniformly reduces nitrogen uptake. Instead, the available evidence supports a more selective response determined by the interaction among yeast physiological state, nitrogen-source composition, and fermentation environment.

The compound-specific nature of the observed changes may reflect several interacting processes. Brewing yeast regulates individual amino acids differently according to transporter specificity, nitrogen-source preference, intracellular biosynthetic demand, and competition among available nitrogen compounds. In addition, repeated harvesting, cold storage, and repitching can alter the physiological state of the yeast population, while modest differences in initial wort composition may influence the relative use of individual nitrogen sources. These factors provide plausible explanations for why selected amino acids may show different trajectories despite stable overall fermentation performance. However, transporter activity, intracellular amino acid pools, gene expression, peptide utilization, and population structure were not measured in the present study. These explanations should therefore be regarded as hypotheses rather than demonstrated mechanisms.

From a technological perspective, an important outcome is that these biochemical changes were not associated with detectable deterioration in product quality. No obvious sensory-quality deterioration was detected by the qualitative quality-control assessment; however, subtle differences among cycles cannot be excluded. Nevertheless, amino acids serve as key precursors for higher alcohols, esters, and other aroma-active compounds, and even small changes in their availability may influence flavor formation. Because volatile metabolites were not analyzed in this study, potential effects on aroma composition cannot be excluded.

Several limitations of the study design should be acknowledged. Each repitching cycle represents a single industrial fermentation rather than an independent biological replicate, and the statistical analyses therefore describe within-process variability rather than population-level reproducibility. In addition, differences in fermentation duration and sampling density between early and later repitching cycles may have influenced the observed trends. For these reasons, the results should be interpreted as exploratory process-level observations rather than confirmatory evidence.

Despite these limitations, the study provides valuable insight into nitrogen metabolism during industrial scale serial repitching. The results indicate that amino acid profiling can serve as a sensitive tool for detecting subtle biochemical changes that are not captured by conventional fermentation metrics. Future studies integrating amino acid analysis with targeted metabolomics, intracellular measurements, and transcriptomic or proteomic approaches will be essential to elucidate the mechanistic basis of these observations and to determine their implications for flavor formation and process optimization.

These findings also complement our previous investigation of carbohydrate utilization during twelve consecutive industrial repitching cycles. Whereas serial repitching altered the dynamics of fermentable sugar utilization, the present results demonstrate that amino acid assimilation remained substantially more conserved, with only subtle, amino acid-specific changes observed across generations. Together, these studies suggest that brewing yeast exhibits distinct process-level patterns in carbon and nitrogen metabolism during prolonged industrial reuse [14].

## 5. Conclusions

Across the twelve consecutive industrial lager fermentations investigated in this study, final alcohol concentration, RDF, apparent extract, and pH remained comparatively stable, indicating the preservation of overall fermentation performance within the observed production sequence. Amino acid profiles changed predominantly with fermentation progression, and the overall depletion pattern remained broadly similar among cycles.

At the level of individual amino acids, aspartate showed the clearest positive association between terminal relative concentration and cycle number and remained statistically supported after correction for multiple testing. Nominal trends for asparagine and serine did not remain significant after FDR correction. Most amino acids showed no clear cycle-associated terminal trend. These findings therefore support selective rather than generalized variation in amino acid utilization across the sequence.

Terminal suspended yeast cell concentrations decreased substantially across cycles, although this trend was not accompanied by a deterioration in final alcohol production or attenuation. Further physiological measurements are required to establish whether this observation is related to yeast adaptation, flocculation behavior, viability, or other process factors.

The twelve fermentations represent one consecutive industrial repitching sequence rather than independently replicated generation treatments, and cycle number is therefore confounded with chronological fermentation order. Accordingly, the observed associations should be interpreted as exploratory process-level trends and not as definitive causal effects of serial repitching. Replicated industrial sequences, together with absolute wort nitrogen profiles, yeast physiological measurements, and analysis of aroma-active metabolites, will be required to establish the reproducibility and mechanistic significance of these observations.

## Figures and Tables

**Figure 1:**
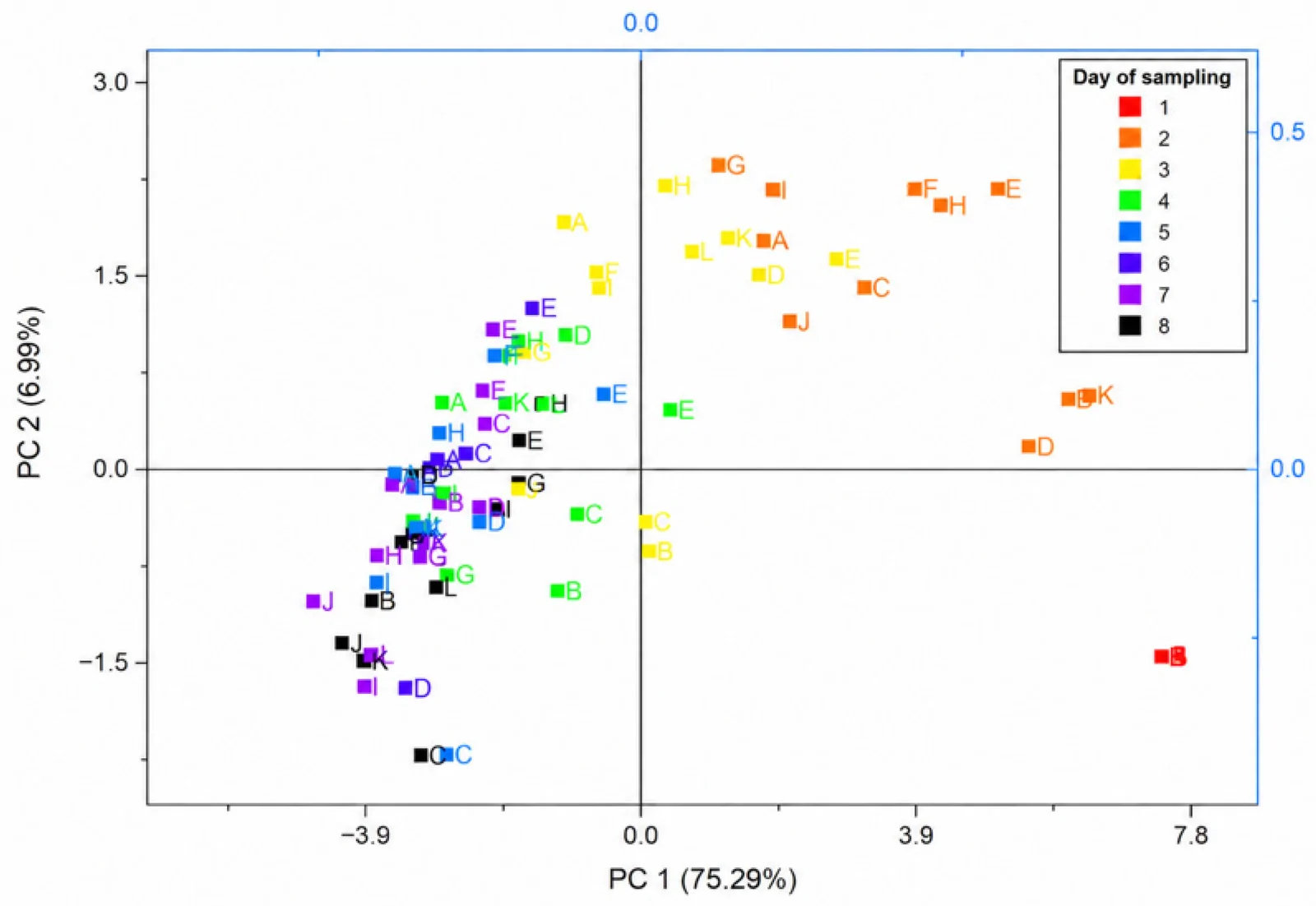
Principal component analysis (PCA) of amino acid profiles. Letters indicate repitching cycles (A–L), and colors indicate the day of sampling.

**Figure 2:**
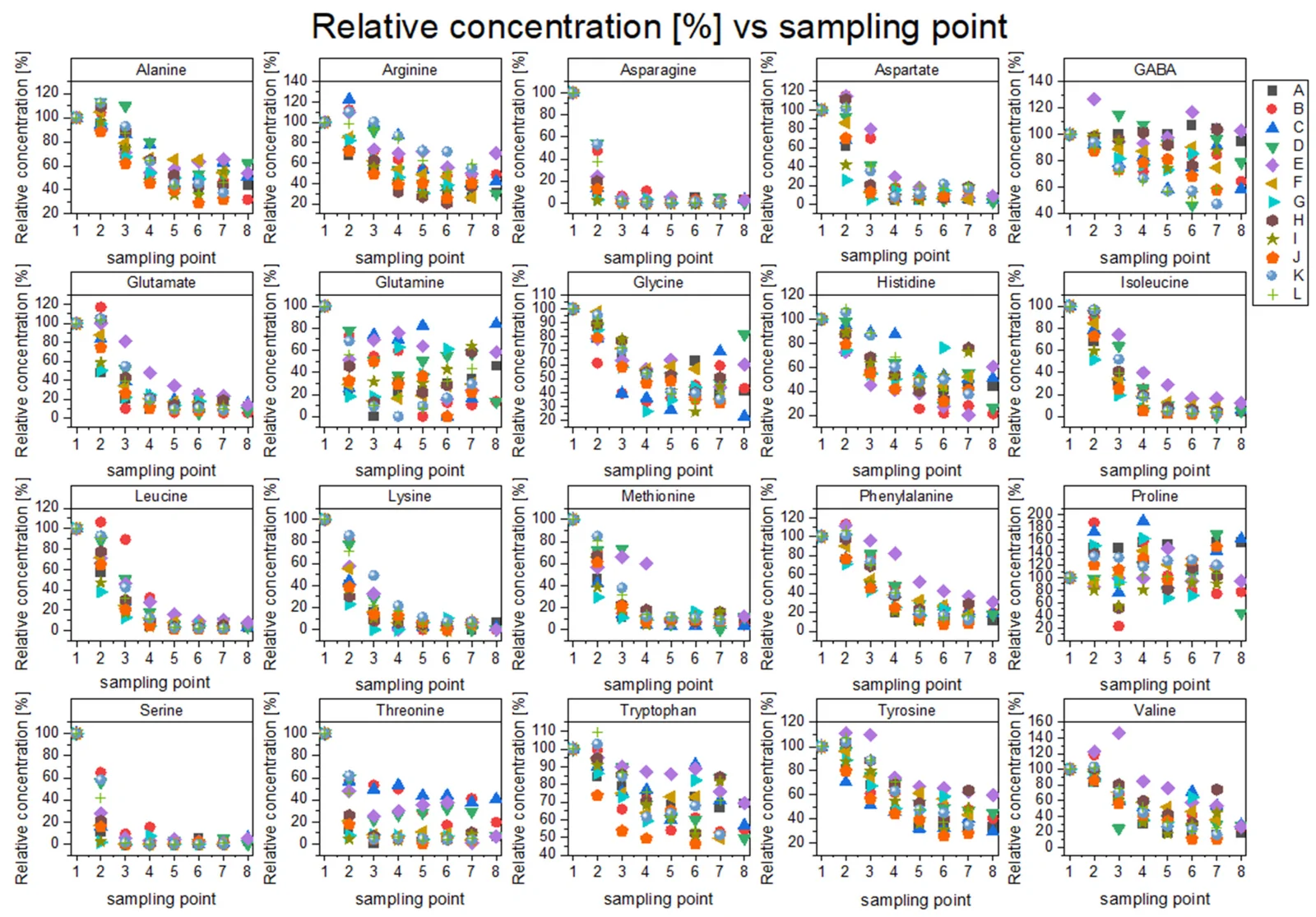
Relative concentrations of amino acids during serial repitching.

**Table 1:** Final beer parameters during serial repitching.

Repitch	Final Apparent Extract (%)	Final Alcohol (vol%)	Final RDF (%)	Final pH	Final Yeast Cells (10^6^/mL)
A	1.80	5.01	83.98	4.32	13.4
B	1.76	5.09	84.50	4.32	13.6
C	1.94	5.07	83.08	4.26	8.9
D	1.92	5.10	83.35	4.38	11.2
E	1.95	5.13	83.22	4.42	9.3
F	1.90	5.12	83.54	4.36	9.8
G	1.87	5.13	83.76	4.26	11.4
H	1.86	5.11	83.78	4.27	6.6
I	1.78	5.04	84.25	4.28	6.3
J	1.81	5.03	84.02	4.26	5.4
K	1.91	4.99	83.14	4.32	4.9
L	1.91	4.99	83.15	4.24	4.4

**Table 2:** Terminal relative amino acid concentrations in %.

		Terminal Relative Amino Acid Concentrations [%]																		
Gen.	Ala	Arg	Asn	Asp	GABA	Glu	Gln	Gly	His	Ile	Leu	Lys	Met	Phe	Pro	Ser	Thr	Trp	Tyr	Val
A	43.9	30.8	2.7	6.7	94.7	12.3	45.2	40.6	43.6	4.3	2.5	6.8	9.1	10.9	155.7	2.8	6.4	69.1	33.3	17.2
B	31.9	48.6	3.2	7.4	64.4	5.4	13.4	43.1	21.2	5.4	3.7	0.0	4.1	19.2	77.2	4.1	19.8	54.4	40.4	25.3
C	51.1	41.9	3.2	4.9	58.9	15.4	83.8	22.8	50.9	5.5	3.5	1.9	3.8	18.7	161.5	6.1	40.8	56.9	29.3	28.7
D	61.9	29.9	0.0	2.6	78.8	7.4	13.0	81.4	26.3	4.9	3.5	0.0	11.1	17.6	44.0	0.0	7.2	49.6	44.9	28.1
E	54.0	69.8	2.4	9.1	103.0	13.9	57.9	60.1	60.6	12.2	7.7	0.0	12.0	31.0	95.0	5.1	6.7	69.4	59.8	26.7
F	54.3	26.3	0.0	5.2	74.9	13.9	29.4	35.4	51.7	7.7	4.3	4.2	11.5	17.3	113.8	0.0	3.7	49.5	43.6	35.3
G	49.0	38.1	0.0	14.7	85.6	17.5	61.1	43.8	76.2	4.1	4.8	10.4	16.2	23.1	71.5	0.0	7.2	82.3	58.8	64.2
H	44.0	36.9	0.0	18.4	103.6	18.6	58.4	50.8	75.9	4.3	5.0	6.5	16.1	28.9	102.2	0.0	10.3	84.1	63.7	74.2
I	55.5	41.4	0.0	15.7	87.6	11.5	63.9	44.4	72.5	2.8	2.8	8.2	15.7	14.4	90.1	0.0	8.7	81.8	48.6	43.9
J	32.4	39.9	0.0	15.8	58.2	7.2	22.1	32.6	41.3	2.5	1.1	5.8	7.6	8.0	148.8	0.0	3.2	51.8	28.1	10.1
K	37.6	54.9	0.0	16.8	47.5	8.2	29.5	35.4	37.6	3.0	1.7	6.7	8.3	12.0	119.6	0.0	3.8	51.6	34.9	16.1
L	46.5	59.2	1.1	20.2	59.3	10.2	43.2	39.6	51.9	3.9	2.3	5.5	9.4	17.2	106.2	1.4	5.4	70.8	46.3	25.2

**Table 3:** Adjusted R^2^, nominal *p*-values, and Benjamini–Hochberg FDR-adjusted q-values for linear regressions of terminal amino acid concentrations against repitching cycle number.

	R^2^	*p*-Value	Benjamini–Hochberg FDR Adjusted *p*-Values (q)
Alanine	−0.07	0.621	0.831
Arginine	0.01	0.314	0.571
Asparagine	0.42	0.013	0.130
Aspartate	0.71	<0.001	<0.020
GABA	0.02	0.298	0.571
Glutamate	−0.10	0.869	0.966
Glutamine	−0.10	0.969	0.969
Glycine	−0.06	0.571	0.831
Histidine	0.02	0.298	0.571
Isoleucine	0.07	0.200	0.571
Leucine	0.03	0.277	0.571
Lysine	0.18	0.092	0.460
Methionine	0.02	0.304	0.571
Phenylalanine	−0.07	0.623	0.831
Proline	−0.10	0.963	0.969
Serine	0.30	0.037	0.247
Threonine	0.14	0.121	0.484
Tryptophan	−0.06	0.568	0.831
Tyrosine	−0.08	0.668	0.835
Valine	−0.09	0.798	0.939

## Data Availability

The original contributions presented in the study are included in the article, further inquiries can be directed to the corresponding author.

## Supplementary Materials

The following supporting information can be downloaded at: https://www.mdpi.com/article/10.3390/foods15193373/s1, Table S1: Initial amino acid concentrations [mg/L]. Table S2: Viability of yeast cells. Table S3: Linear mixed-effects model results for the effect of sampling day on relative amino acid concentrations during twelve consecutive repitching cycles.

## Abbreviations

The following abbreviations are used in this manuscript:

FAN	Free amino nitrogen
HPLC	High-performance liquid chromatography
FLD	Fluorescence detector
OPA	o-Phthalaldehyde
Ala	Alanine
Arg	Arginine
Asn	Asparagine
Asp	Aspartate
Cys	Cysteine
Glu	Glutamate
Gln	Glutamine
Gly	Glycine
His	Histidine
Ile	Isoleucine
Leu	Leucine
Lys	Lysine
Met	Methionine
Phe	Phenylalanine
Pro	Proline
Ser	Serine
Thr	Threonine
Trp	Tryptophan
Tyr	Tyrosine
Val	Valine
