# Supplementary material for: Amino Acid Utilization During Twelve Consecutive Industrial Repitching Cycles of Saccharomyces pastorianus in Lager Beer Fermentation
Source: Foods. 2026 Sep 22;15(19):3373. doi: 10.3390/foods15193373 (PMC13664451; doi:10.3390/foods15193373)
Supplement: Supplementary file 1 [file foods-15-03373-s001.zip › foods-4551414-supplementary.pdf]

| | Initial amino acid concentrations [mg/L] | | | | | | | | | | | | | | | | | | | |
|---|---|---|---|---|---|---|---|---|---|---|---|---|---|---|---|---|---|---|---|---|
| **Gen.** | **Ala** | **Arg** | **Asn** | **Asp** | **GABA** | **Glu** | **Gln** | **Gly** | **His** | **Ile** | **Leu** | **Lys** | **Met** | **Phe** | **Pro** | **Ser** | **Thr** | **Trp** | **Tyr** | **Val** |
| A | 71,2 | 113,4 | 71,0 | 44,2 | 94,4 | 38,8 | 8,7 | 27,2 | 55,7 | 51,9 | 119,0 | 79,5 | 30,2 | 96,6 | 190,5 | 42,5 | 44,8 | 29,0 | 71,9 | 83,2 |
| B | 84,3 | 100,0 | 56,6 | 73,4 | 161,1 | 68,1 | 26,0 | 27,5 | 103,5 | 70,0 | 97,6 | 77,0 | 26,6 | 121,5 | 315,0 | 37,9 | 35,2 | 46,7 | 91,8 | 106,1 |
| C | 72,8 | 85,3 | 85,1 | 93,2 | 168,0 | 60,5 | 15,0 | 27,9 | 70,6 | 74,8 | 134,6 | 108,8 | 38,7 | 157,0 | 316,7 | 65,3 | 35,9 | 42,5 | 129,8 | 129,0 |
| D | 69,6 | 140,0 | 97,8 | 99,8 | 123,6 | 77,6 | 27,9 | 17,2 | 87,0 | 67,9 | 122,9 | 94,8 | 28,0 | 132,4 | 529,3 | 73,8 | 61,2 | 45,5 | 86,7 | 122,7 |
| E | 82,6 | 86,2 | 78,5 | 55,6 | 93,2 | 47,0 | 14,6 | 14,5 | 124,6 | 49,8 | 113,3 | 91,6 | 24,1 | 94,4 | 304,3 | 58,5 | 46,9 | 27,3 | 68,7 | 79,8 |
| F | 79,6 | 99,4 | 74,5 | 54,0 | 114,9 | 44,8 | 13,4 | 29,1 | 57,9 | 57,8 | 128,7 | 91,8 | 34,8 | 108,0 | 229,1 | 47,1 | 49,2 | 33,0 | 79,8 | 86,5 |
| G | 75,5 | 92,4 | 68,7 | 49,7 | 120,6 | 39,6 | 11,3 | 28,4 | 57,3 | 53,9 | 120,3 | 90,3 | 32,6 | 99,1 | 250,5 | 47,2 | 45,8 | 32,4 | 75,0 | 82,0 |
| H | 68,0 | 85,6 | 54,1 | 37,0 | 101,8 | 33,5 | 12,1 | 21,5 | 58,7 | 42,9 | 103,2 | 128,4 | 30,6 | 74,5 | 215,8 | 42,5 | 34,7 | 29,3 | 67,5 | 68,2 |
| I | 67,4 | 58,5 | 58,0 | 41,8 | 109,2 | 35,8 | 9,0 | 19,5 | 46,4 | 45,0 | 102,6 | 94,0 | 26,3 | 76,6 | 256,3 | 39,9 | 35,0 | 26,2 | 64,8 | 86,9 |
| J | 95,8 | 85,9 | 69,1 | 66,6 | 120,1 | 45,1 | 15,0 | 21,0 | 81,0 | 54,8 | 162,6 | 97,6 | 43,0 | 99,2 | 204,8 | 64,1 | 55,8 | 37,7 | 91,5 | 122,8 |
| K | 81,2 | 57,7 | 67,1 | 57,2 | 128,0 | 39,4 | 13,1 | 18,5 | 78,3 | 46,3 | 141,3 | 101,1 | 35,0 | 86,1 | 237,6 | 50,2 | 47,1 | 30,6 | 73,3 | 104,1 |
| L | 85,3 | 66,7 | 70,0 | 53,8 | 127,7 | 40,5 | 13,0 | 19,7 | 77,4 | 44,6 | 146,0 | 96,6 | 34,3 | 87,5 | 296,9 | 50,6 | 48,4 | 28,7 | 74,2 | 88,3 |

*Supplementary Table S1: Initial amino acid concentrations [mg/L]*

| Starter culture of yeast | Viability of yeast cells [%] |
|---|---|
| A | 98±1,0 |
| B | 97±1,0 |
| C | 97±1,0 |
| D | 98±1,0 |
| E | 96±1,0 |
| F | 98±1,0 |
| G | 95±1,0 |
| H | 98±1,0 |
| I | 97±1,0 |
| J | 98±1,0 |
| K | 96±1,0 |
| L | 95±1,0 |

*Supplementary Table S2: Viability of yeast cells*

| Amino acid | N observations | N cycles | Overall Day effect: Wald $\chi^2$ | df | P-value | FDR-adjusted q-value | Cycle random-intercept variance | Residual variance | ICC | Converged |
|---|---|---|---|---|---|---|---|---|---|---|
| Alanine | 88 | 12 | 425,63 | 7 | <0,001 | <0,001 | 43,19 | 94,43 | 0,314 | Yes |
| Arginine | 88 | 12 | 375,18 | 7 | <0,001 | <0,001 | 146,96 | 109,59 | 0,573 | Yes |
| Asparagine | 88 | 12 | 2022,28 | 7 | <0,001 | <0,001 | 3,48 | 48,87 | 0,066 | Yes |
| Aspartate | 88 | 12 | 624,98 | 7 | <0,001 | <0,001 | 45,47 | 176,25 | 0,205 | Yes |
| GABA | 88 | 12 | 46,29 | 7 | <0,001 | <0,001 | 103,07 | 132,37 | 0,438 | Yes |
| Glutamate | 88 | 12 | 820,07 | 7 | <0,001 | <0,001 | 33,04 | 125,82 | 0,208 | Yes |
| Glutamine | 88 | 12 | 80,55 | 7 | <0,001 | <0,001 | 0,00 | 579,98 | 0,000 | Yes |
| Glycine | 88 | 12 | 129,75 | 7 | <0,001 | <0,001 | 2,00 | 255,75 | 0,008 | Yes |
| Histidine | 88 | 12 | 182,91 | 7 | <0,001 | <0,001 | 12,29 | 204,56 | 0,057 | Yes |
| Isoleucine | 88 | 12 | 2015,98 | 7 | <0,001 | <0,001 | 39,66 | 56,90 | 0,411 | Yes |
| Leucine | 88 | 12 | 1203,57 | 7 | <0,001 | <0,001 | 37,10 | 92,39 | 0,286 | Yes |
| Lysine | 88 | 12 | 1179,77 | 7 | <0,001 | <0,001 | 14,85 | 83,47 | 0,151 | Yes |
| Methionine | 88 | 12 | 740,92 | 7 | <0,001 | <0,001 | 16,26 | 121,18 | 0,118 | Yes |
| Phenylalanine | 88 | 12 | 1363,83 | 7 | <0,001 | <0,001 | 81,78 | 66,64 | 0,551 | Yes |
| Proline | 88 | 12 | 22,00 | 7 | 0,003 | 0,003 | 157,73 | 705,85 | 0,183 | Yes |
| Serine | 88 | 12 | 1477,97 | 7 | <0,001 | <0,001 | 5,68 | 66,95 | 0,078 | Yes |
| Threonine | 88 | 12 | 711,02 | 7 | <0,001 | <0,001 | 138,28 | 105,43 | 0,567 | Yes |
| Tryptophan | 88 | 12 | 116,81 | 7 | <0,001 | <0,001 | 70,69 | 142,52 | 0,332 | Yes |
| Tyrosine | 88 | 12 | 239,05 | 7 | <0,001 | <0,001 | 91,08 | 188,98 | 0,325 | Yes |
| Valine | 88 | 12 | 318,96 | 7 | <0,001 | <0,001 | 96,27 | 198,85 | 0,326 | Yes |

*Supplementary Table S3: Linear mixed-effects model results for the effect of sampling day on relative amino acid concentrations during twelve consecutive repitching cycles*
